# Synthesis of Porous Hollow Spheres Co@TiO_2−*x*_-Carbon Composites for Highly Efficient Lithium-Ion Batteries

**DOI:** 10.1186/s11671-022-03719-y

**Published:** 2022-09-05

**Authors:** Chunyong Liang, Zhongliang Huang, Hongshui Wang, Tai Yang, Ning Liu, Tingdi Liao, Feng Wang, Xi Wang

**Affiliations:** 1 Fujian Provincial Key Laboratory for Advanced Micro-Nano Photonics Technology and Devices, Fujian Provincial Collaborative Innovation Center for Ultra-Precision Optical Engineering and Applications, Quanzhou Normal University, Quanzhou, 362000 Fujian China; 2 State Key Laboratory of Reliability and Intelligence of Electrical Equipment, School of Materials Science and Engineering, Hebei University of Technology, Tianjin, 300130 China; 3 College of Physics and Information Engineering, Quanzhou Normal University, Quanzhou, 362000 Fujian China; 4 China Center for Information Industry Development, Beijing, 100048 China; 5 Changzhou Blon Minimally Invasive Medical Devices Technology Co. Ltd., Changzhou, 213100 Jiangsu China

**Keywords:** Lithium-ion batteries, Titanium dioxide, Oxygen vacancies, Chemical vapor deposition

## Abstract

The hollow TiO_2_ anode material has received great attention for next-generation LIBs because of its excellent stability, environmental friendly, and low volume change during lithiation/delithiation. However, there are some problems associated with the current anatase TiO_2_ anode materials in practical application owing to low lithium-ion diffusivity and poor reversible theoretical capacities. The introduction of defects has been turned out to be a significant and effective method to improve electronic conductivity, especially oxygen vacancies. In this paper, a facile hydrothermal reaction and subsequent chemical vapor deposition method were successfully used to fabricate Co@TiO_2−*x*_-carbon hollow nanospheres. These results suggest that the synthesized product exhibits good rate performance and superior cycling stability.

## Introduction

Lithium-ion battery has high energy storage and long cycle life, so it has a great research and application value secondary battery field [1, 2]. In general, graphite is used as the anode of the Lithium-ion battery. However, it has a relatively low theoretical gravimetric capacity, which cannot achieve the demand of LIBs industry for high theoretical capacity and energy density. Therefore, a variety of alternative anode materials have been widely studied [3–5]. Among all proposed candidates for the anode, TiO_2_ shows the advantages of high theoretical capacity (335 mAh g^−1^), excellent Li-ion storage performances and environmental friendliness. In addition, the hollow nanospheres structure of TiO_2_ provided a sufficient amount of space to inhibit the volume change effectively during the lithiation/delithiation process. Therefore, TiO_2_ has become promising alternative anode materials for high-safety LIBs. However, the poor conductivity of TiO_2_ causes the large initial impedance of TiO_2_ anode, which seriously affects its rate performance [6–9]. In order to solve the inherent defect, different methods have been tried in recent years, including reducing particle size, doping elements, carbon coating, designing nanostructure morphology and introducing defects [10, 11].

In these methods, the introduction of defects has been proved to be effective in improving electronic conductivity, especially in the production of OVs, which is self-modifying and easy to operate [12]. The methods of introducing OVs into TiO_2_, including hot hydrogen, plasma, electrochemical and deoxidizer hydrothermal reductions reactions [13, 14]. The introduction of nanostructures and conductive materials can also improve the electrochemical performance of TiO_2_ anode [15, 16]. The high specific surface of the hollow nanospheres structure can fast highways for lithium ion (Li^+^) and improve the conductivity of LIBs. Moreover, the introduction of conductive materials can also improve the conductivity and buffer volume expansion during lithiation and delithiation. Both of them enhanced the electrochemical performances of the TiO_2_ anode [17].

Inspired by this, we prepared Co@TiO_2−*x*_-carbon hollow nanospheres by hydrothermal method and chemical vapor deposition (CVD) method to obtain excellent LIBs anode materials. Smooth conformal amorphous carbon with controllable thickness was deposited on the active material by CVD, which significantly improves the electrochemical performance of active materials. The optimized Co@TiO_2−*x*_-carbon composite has porous, rich defects and carbon coating properties. Carbon coating, metal cobalt and oxygen vacancies can increase the electrical conductivity of the materials, thus improving the rate performance; porous hollow nanospheres can provide more lithium storage sites, shorter Li^+^ and electron diffusion path, at the same time, the appropriate internal cavity can inhibit the volume expansion during the cycle, so it has outstanding rate ability and cycling performance [18]. Consequently, the composite electrode presents a favorable rate performance with high current density (217 mAh g^−1^ at 1 A g^−1^). Even after 1000 long cycles, a high capacity of 173 mAh g^−1^ at 1 A g^−1^ can also be achieved, which shows an excellent cycle performance.

## Materials and Methods

### Material Preparation

#### Synthesis of TiO_2_

The TiO_2_ microspheres were prepared through hydrothermal method. Ti(SO_4_)_2_·9H_2_O (0.81 g) was uniformly dissolved in anhydrous ethanol (75 mL) to form a homogeneous solution, denoted as solution A. Next, 6 mL H_2_O_2_ and 0.43 g NH_4_Cl were added to the above solution to make clear solution with continuous stirring (solution B). The obtained solution B was transferred to an autoclave, followed by a hydrothermal treatment in an oven at 120 °C for 12 h. The white precipitated product was separated by centrifugation and washed with deionized water and absolute ethanol, subsequently dried in an oven.

#### Synthesis of Co@TiO_2−*x*_-Carbon Composite

**Fig. 1:**
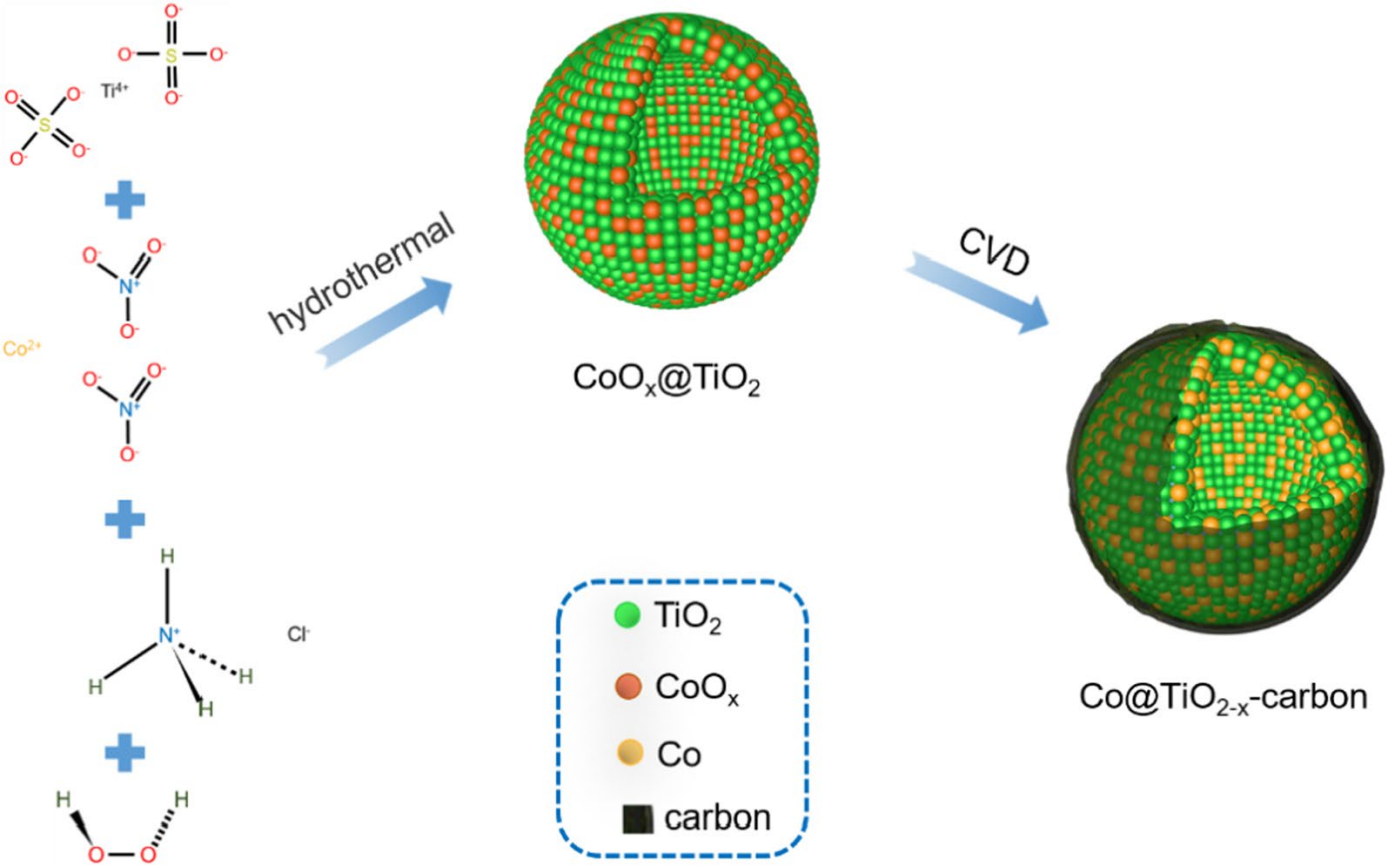
Synthesis strategy of hollow Co@TiO_2−*x*_-carbon composite

Firstly, 0.04 g Co(NO_3_)_2_·6H_2_O materials were dissolved in the mixed solution B and then according to the above method, the CoO_*x*_@TiO_2_ material was obtained. The Co@TiO_2−*x*_-carbon composite was synthesized by a subsequent annealed treatment. The as-formed CoO_*x*_@TiO_2_ composite was put in a ceramic boat and heated to 350 °C in the tube furnace under Ar atmosphere, and further heated to 560 °C under H_2_ atmosphere; Finally, the powder is calcined at 560 °C for 30 min in C_2_H_2_ atmosphere. The synthesized sample was named as Co@TiO_2−*x*_-carbon. Figure1 illustrates the flowchart for the preparation of the composite material.

### Material Preparation

Phase compositions of samples were determined by X-ray diffraction (XRD) using Bruker D8 Discover diffractometer. Scanning electron and transmission electron microscopies (SEM and TEM) images were obtained by using Hitachi S-4800 and JEM 2100F (JEOL) instruments, respectively. The elemental analysis was performed using the Axis Ultra DLD spectrometer. The nitrogen adsorption-desorption isotherms and Brunauer-Emmett-Teller (BET) surface area were determined by the V-Sorb 2800P instrument. X-ray photoelectron spectroscopy (XPS, K-Alpha XPS) and electron paramagnetic response (EPR, Bruker A300) were used to characterize chemical states of the samples.

### Electrochemical Measurements

At room temperature, a coin cell (CR2032) was used to conduct the electrochemical test with as-prepared materials as an anode. The as-prepared material, conductive agent super P and polyvinylidene fluoride (PVDF) were mixed with NMP at the mass ratio of 8:1:1 to obtain the slurry. Subsequently, the slurry was coated on a copper foil with a scraper, and then dried in a vacuum to form the electrode plate. The anode was obtained by punching a circle with a diameter of 12 cm. Coin-type cells assembled in a glove box filled with Ar gas consisted of the prepared electrodes, a Celgard membrane as a separator, metallic Li foils as counter electrodes. The electrolyte is composited of 1.0 M LiPF_6_ in ethylene carbon (EC)/dimethyl carbonate (DMC) (1:1 w/w). Cyclic voltammetry (CV) curves were recorded at 0.1 mV s^-1^ between 0.01 and 3.0 V via an electrochemical workstation (CHI760E). The charge/discharge curves of electrode material at different rate test was conducted with the voltage range from 0.01 to 3.0 V.

## Results

**Fig. 2:**
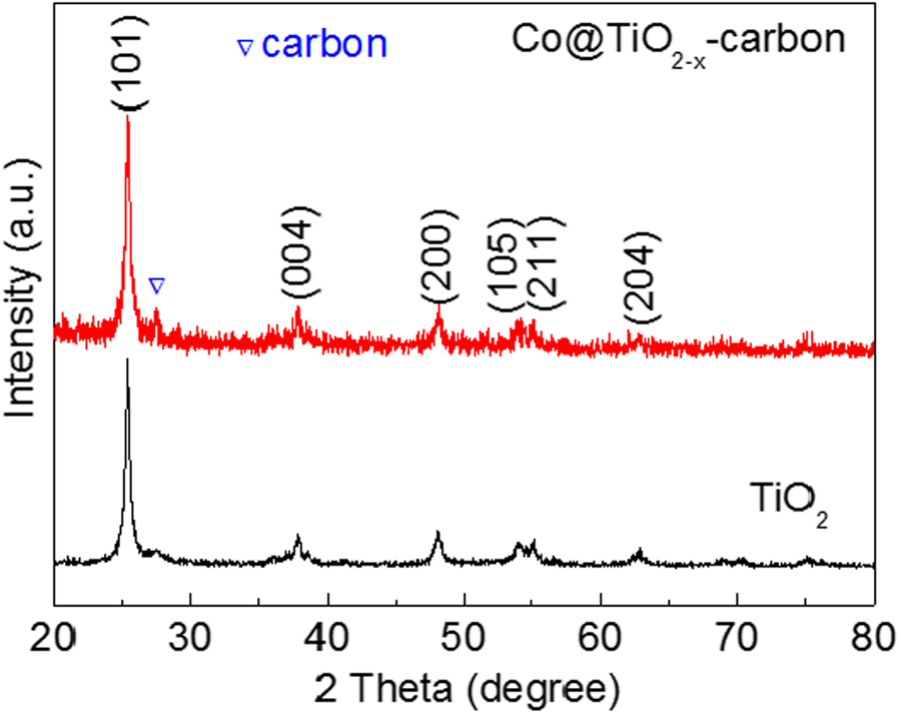
XRD pattern of the synthesized samples

To verify the crystalline structure of the synthesized products, the XRD patterns are presented in Fig. 2. The characteristic peaks at 25.3°, 37.8° and 48° correspond to (101), (004) and (200) planes of TiO_2_ (PDF#78-2486), respectively, indicating that the synthesized products have a high crystalline nature. The wide peak of Co@TiO_2−*x*-_carbon at about 26.7° indicates the success of carbon coating [19]. In the figure no other impurities are detected, confirming that the as-prepared products have high purity. Therefore, XRD showed that Co@TiO_2−*x*_-carbon and TiO_2_ were successfully fabricated.

**Fig. 3:**
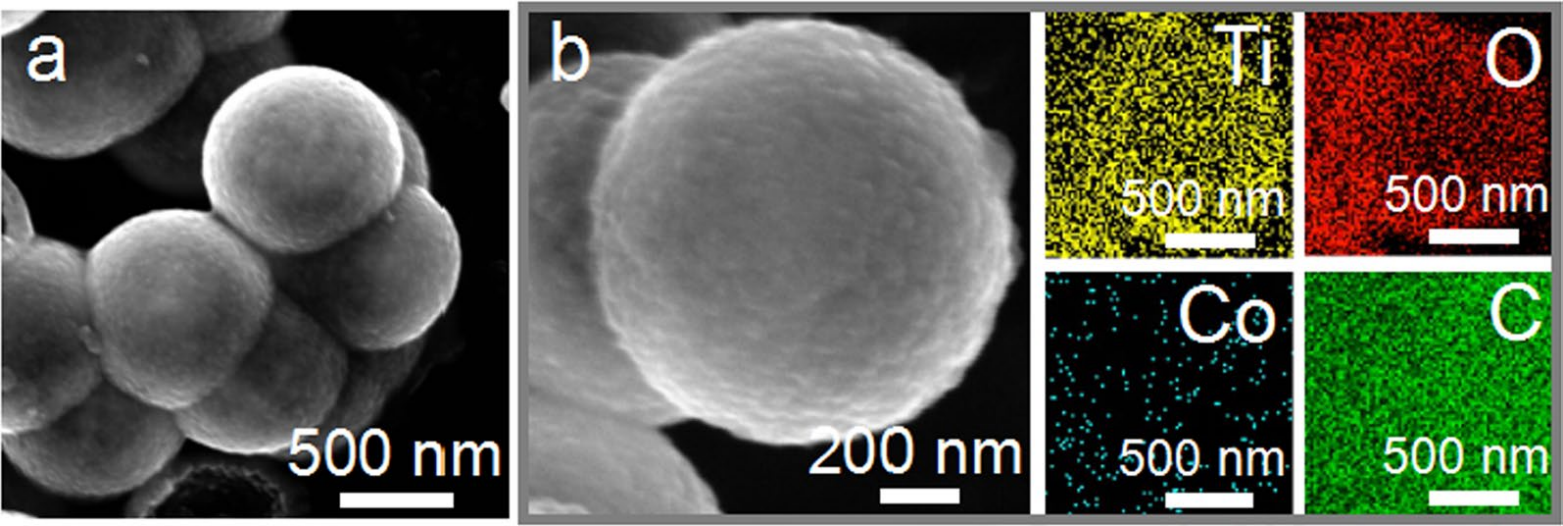
**a** SEM image; **b** high-resolution SEM image and corresponding EDS mappings of Co@TiO_2−*x*_-carbon

The characterization analysis about the basic morphological structure of Co@TiO_2−*x*_-carbon nanoparticles was conducted by SEM. It is observed that the hollow composite possessed a nearly homogeneous spherical shape and a high dispersity. (Fig. 3a). The diameter of the nanospheres is about 800 nm. As shown in Fig. 3b, Ti, O, Co and C are uniformly dispersed on the nanospheres.

**Fig. 4:**
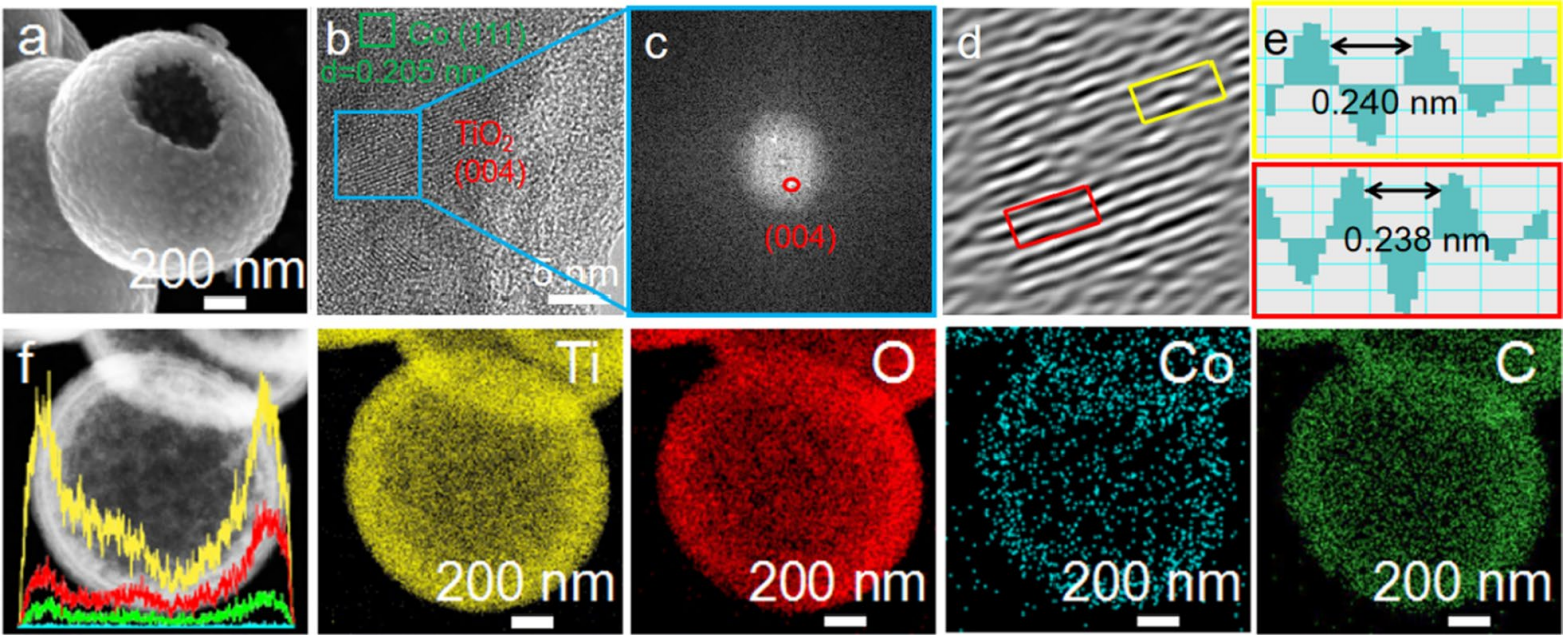
**a** SEM; **b** high-resolution TEM image; **c** FFT pattern; **d** the inverse FFT crystalline lattice image; **e** the lattice spacing profiles at selected areas in yellow and red; **f** linear scan and corresponding elemental mappings of Co@TiO_2−*x*_-carbon

The SEM image reveals that the as-synthesized composite forms a hollow nanospheres structure in Fig. 4a. Meanwhile, the hollow morphology of the composite has been further described by the TEM images in Fig. 4f. The porous hollow spherical structure can provide more lithium storage sites, shorter lithium ion and electron diffusion paths. At the same time, the appropriate internal cavity can well adjust the structure and volume change of the electrode. The HRTEM of Fig. 4b shows the existence of cobalt metal and TiO_2_. The existence of TiO_2_ can be further clearly observed by FFT and inverse FFT transformation of Fig. 4c–e. The existence of Ti^3+^ and oxygen vacancies can facilitate the migration of electron of the materials; thus, the composite electrode exhibits a superior rate performance. The linear scanning and corresponding element mapping of Fig. 4f show that TiO_2_ and cobalt metal are uniformly distributed on the surface of hollow nanospheres.

**Fig. 5:**
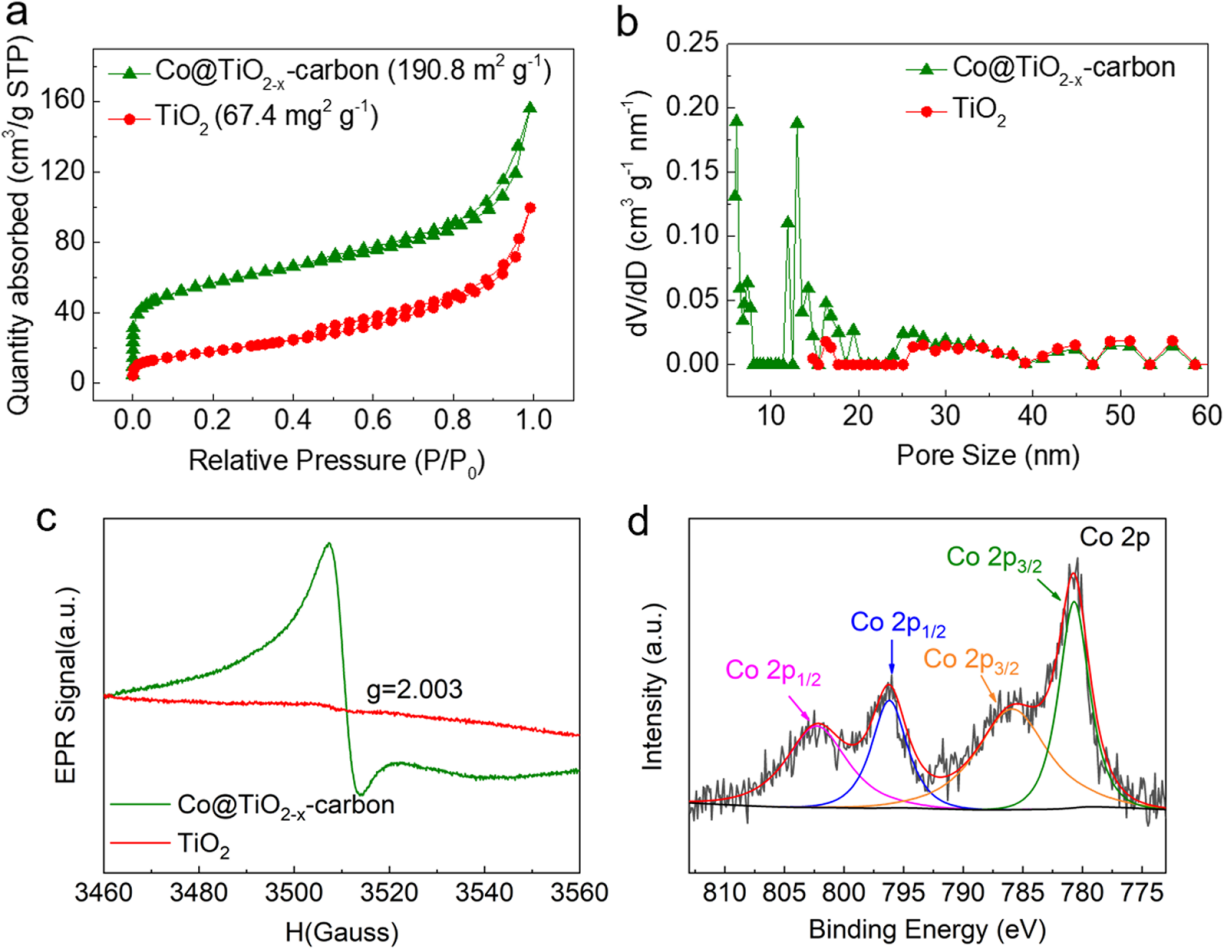
**a** N_2_ adsorption-desorption isotherms; **b** pore size distributions; **c** EPR spectra of Co@TiO_2−*x*_-carbon and TiO_2_; **d** XPS spectra of Co@TiO_2−*x*_-carbon

A nitrogen adsorption experiment was used to test the pore characteristics of synthetic products, as shown in Fig. 5a. Compared with TiO_2_ (67.4 m^2^ g^−1^), the composite possesses a high specific surface area which was about 190.8 m^2^ g^−1^. The N_2_ adsorption-desorption isotherm curves of TiO_2_ and Co@TiO_2−*x*_-carbon belong to type-IV isotherm, indicating abundant mesopore structure (Fig. 5b). Co@TiO_2−*x*_-carbon composite has a wide range of mesoporous size distribution, which is conducive to the transport of lithium ions and can accommodate huge volume changes in the cycling process. Besides, the abundant mesoporous structure and large specific surface area provide more lithium insertion sites and voids to facilitate the storage of lithium and improve the rate ability and cycling performance of LIBs. EPR was employed to characterize the chemical states in TiO_2_ and Co@TiO_2−*x*_-carbon. The strong EPR signal obtained from Co@TiO_2−*x*_-carbon with a g-value of 2.003 indicates the existence of oxygen vacancies, while only a trace amount of oxygen vacancies was observed for pristine TiO_2_ (Fig. 5c). XPS spectra of Co@TiO_2−*x*_-carbon composites were recorded and analyzed. These peaks correspond to Co 2p_3/2_ and Co 2p_1/2_, respectively, and suggest that the reduction in Co 2p in Co(NO_3_)_2_·6H_2_O produced Co nanoparticles (Fig. 5d).

**Fig. 6:**
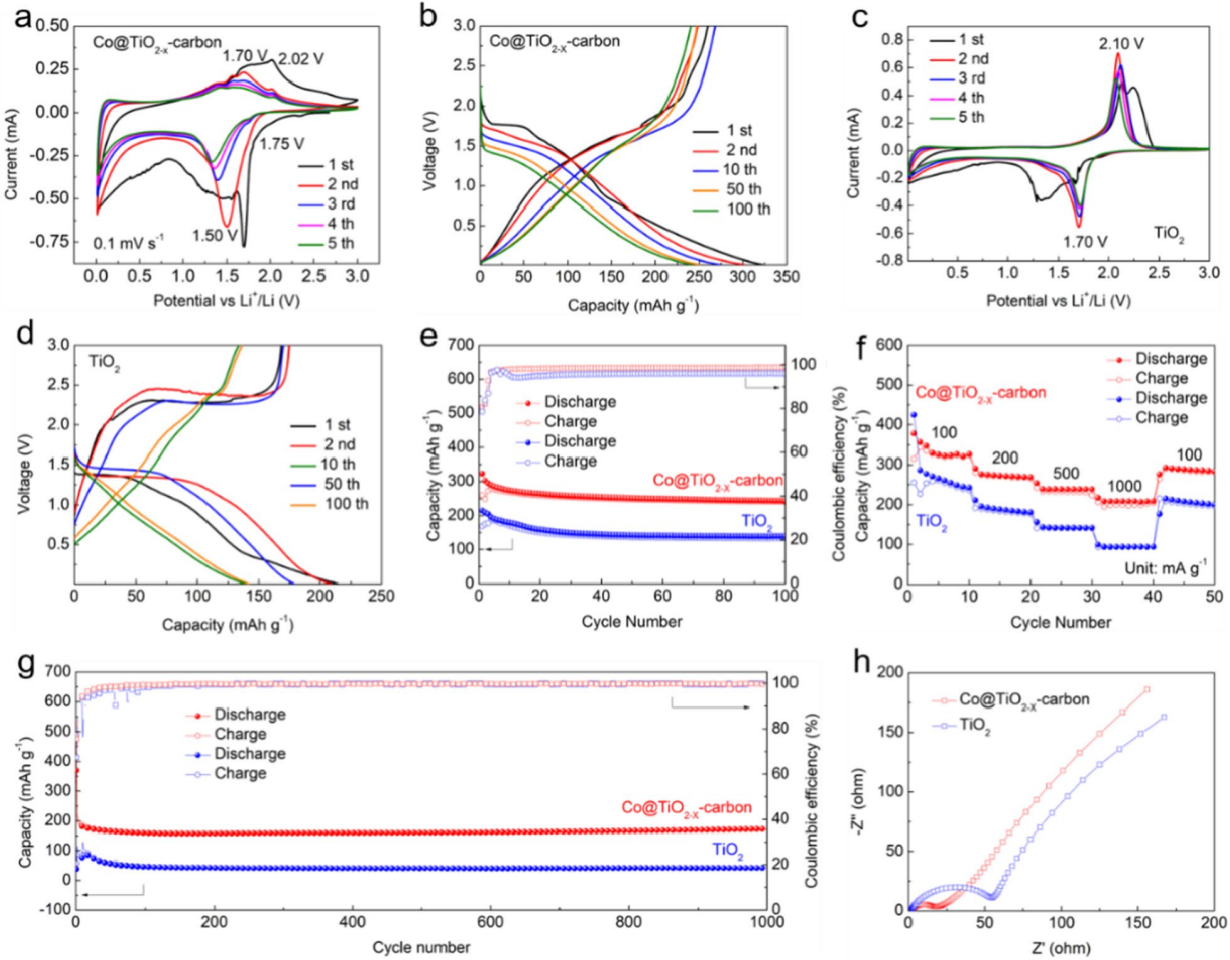
CV curves of **a** Co@TiO_2−*x*_-carbon and **c** TiO_2_; Charge–discharge voltage profiles of **b** Co@TiO_2−*x*_-carbon and **d** TiO_2_ at 0.2 A g^−1^; **e** Cyclic stability and **f** rate capabilities of as-synthesized products; **g** Long cycling performance of as-synthesized products at 1 A g^−1^; **h** Nyquist plots of as-synthesized products before cycling

Electrochemical properties of the anode materials were further studied, as shown in Fig. 6, the lithium storage capacity was evaluated by half cells and prepared electrode. Due to the lithiation/delithiation effect of the carbon phase, the range (0.01–3 V) can lead to higher reversible capacity.

The lithium storage behaviors of the synthesized samples were investigated in half cells at a scanning rate of 0.1 mV s^−1^ and in a scanning range of 0.01–3 V. The curves show the initial five cycles of the as-prepared products are shown in Fig. 6a, c. The first small reduction peak of the composite at 1.75 V during the first discharge can be attributed to the insertion of Li^+^ into TiO_2_ to form Li_0.5_TiO_2_ (The inset images in Fig. 6a). As we all known, due to the existence of TiO_2−*x*_, the conductivity of Li_x_TiO_2_ formed in situ is also improved [20, 21]. Meanwhile, in subsequent cycles, the reduction/oxidation peak of 1.50/1.70 can be attributed to TiO_2−*x*_ and the reduction/oxidation peak of 1.75/2.02 can be attributed to TiO_2_ [22]. The CV curves of TiO_2_ sample displays one oxidation peak at approximately 2.10 V and a reduction peak at approximately 1.70 V, which are in agreement with anatase TiO_2_ (Fig. 6c) [23].

Discharge/charge profiles of lithium-ion battery with two samples are exhibited in Fig. 6b, d at 0.2 A g^−1^ under a voltage window of 0.01–3 V. The first discharge capacity of the composite electrode is 323 mAh g^−1^, superior to TiO_2_ electrode (214 mAh g^−1^). Simultaneously, the result of the first cycle shows that the composite electrode exhibits low initial coulombic efficiency (ICE), which may be associated with the construction of a solid electrolyte interface (SEI) membrane [24]. Furthermore, the complex anode delivers a discharge specific capacity of 302 mAh g^−1^ in the following cycle, whereas TiO_2_ electrode in the same process only 209 mAh g^−1^. Compared with TiO_2_ electrode, the charge-discharge curve of the composite electrode shows a similar and clear potential platform and is stable, which reveals the prominent reversibility of electrochemical reactions for the composite anode [25].

As shown in Fig. 6e, Co@TiO_2−*x*_-carbon provides the highest discharge specific capacity during the test at 0.2 A g^−1^. After 100 cycles, the composite material delivers a higher reversible capacity of 242 mAh g^−1^ than TiO_2_ nanoparticles (139 mAh g^−1^), demonstrating that the composite material possesses an outstanding reversibility [26]. The introduction of the carbon layer can increase the capacity of LIBs. In addition, the design of porous hollow nanospheres, the introduction of oxygen vacancies and metal cobalt can promote cycle stability.

The rate capability of the electrodes is further compared in Fig. 6f. Compared with TiO_2_, the electrochemical performance of the composite electrode has a higher reversible capacity and better cycling performance at different current densities. The discharge capacities under the conditions of 0.1, 0.2, 0.5 and 1 A g^−1^ were 379, 291, 253 and 217 mAh g^−1^, respectively. Electron conductivity and diffusion path determine the rate performance of LIBs [27, 28]. The structure of hollow nanospheres of the Co@TiO_2−*x*_-carbon shortens the diffusion path of Li^+^ and electron, and the existence of VOs, carbon coating and cobalt metal improve the conductivity essentially.

It is worth noting that Co@TiO_2−*x*_-carbon exhibits strong cycle stability (no significant capacity degradation and close to 100% coulombic efficiency) at a high current density at 1 A g^−1^ and maintains 173 mAh g^−1^ after 1000 cycles. Obviously, the composite electrode displays uch better cycle performance than TiO_2_, and the reversible specific capacity of the composite material is much higher than that of TiO_2_ (42 mAh g^−1^ at the 1000 th cycle) in Fig. 6g. Due to the specific hollow structure of the as-prepared product, which can inhibit the volume expansion during the cycling process and obtain excellent cycling performance.

Figure 6h shows the EIS of anode materials. The two Nyquist plots are both consisted of a compressed semicircle at the high-frequency region followed by a sloping line in the low frequency region. The low-frequency region represents the Warburg diffusion process which might be attributed to the diffusion of the lithium ions, and the semicircle at high frequency corresponds to the charge-transfer resistance [29]. Clearly, the composite has lower resistance and better conductivity than the TiO_2_ sample, which are caused by the Li-ion insertion/extraction rates. Therefore, the composite product exhibits superior electrochemical reaction kinetics.

## Conclusions

In summary, a simple hydrothermal and CVD method was used to synthesize Co@TiO_2−*x*_-carbon hollow nanospheres. The addition of OVs, Co metal and carbon layer improves the electrical conductivity of the synthesis product to a certain extent, thus exhibiting a remarkable rate performance of the composite. Hollow nanospheres have rich pore size, provide more lithium storage sites, shorter lithium ion and electron diffusion path. At the same time, the appropriate cavity structure can better inhibit the volume change during the cycle, and significantly improve the cycle stability. The results show that Co@TiO_2−*x*_-carbon hollow nanospheres are promising materials for advanced anode materials of LIBs.

## Data Availability

All of the material is owned by the authors and/or no permissions are required.
